# Intestinal gases as markers to study the diet–gut microbiota–host metabolism axis in humans and their relationship to metabolic health

**DOI:** 10.1080/19490976.2026.2701495

**Published:** 2026-07-14

**Authors:** Gillian N.F. Larik, Emanuel E. Canfora, Evert M. van Schothorst, Ellen E. Blaak

**Affiliations:** a Department of Human Biology, School of Nutrition and Translational Research in Metabolism (NUTRIM), Maastricht University, The Netherlands; b Human and Animal Physiology, Wageningen University, Wageningen, The Netherlands

**Keywords:** glucose metabolism, obesity, intestinal bacteria, intestinal gas, fermentation

## Abstract

The gut microbiota‒host metabolism axis is a critical determinant of metabolic health, yet its functional activity remains difficult to monitor *in vivo*. The gut microbiota ferments undigested food components such as dietary fibers and proteins, yielding various important metabolites and gases that impact human metabolism. This review synthesizes current evidence on intestinal gases, primarily hydrogen (H_2_), methane (CH_4_) and hydrogen sulfide (H_2_S), as non-invasive markers of the trade-off between saccharolytic and proteolytic fermentation. Within the current review, we evaluate the concentrations of important gut metabolites and gases and techniques to measure intestinal gases, including stable isotope breath tests, volatile organic compound (VOC) profiling, and respiration chambers. Crucially, we highlight that these gases may function not only as metabolic byproducts and biomarkers of microbial activity but as active signaling molecules influencing gastrointestinal transit, satiety, and systemic inflammation. This review concludes that real-time gas monitoring provides a unique opportunity to study real-time gut microbial fermentation. We propose a framework for phenotyping individual or subgroup-based fermentation patterns to guide personalized nutritional interventions for obesity and type 2 diabetes.

## Introduction

The prevalence of obesity and its related metabolic disturbances, including insulin resistance, metabolic syndrome, and type 2 diabetes (T2D) have increased dramatically over the past decades and is considered a major health problem.^1-3^ These conditions typically manifest because of an increased intake of energy-dense, low-fiber foods and reduced physical activity, leading to a positive energy balance and weight gain. However, the development of obesity has a complex multifactorial etiology where many factors, including environmental, socioeconomic, genetic, and biological factors, are important determinants.^4^ Over the past years, the gut microbiota, particularly the bacterial communities residing in the colon, have emerged as an important regulator of host energy and substrate metabolism and may thus play a crucial role in the etiology of obesity, T2D, and related metabolic disturbances.^5^ ^,^ ^6^

**Figure 1.**
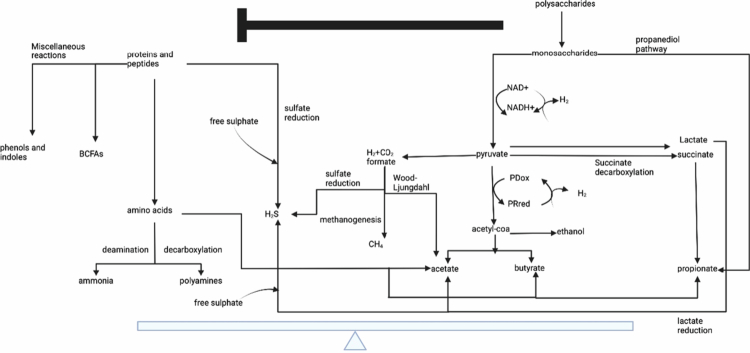
Microbial pathways of saccharolytic and proteolytic fermentation. This figure provides an overview of the major microbial metabolic pathways, intermediary and end-products of both saccharolytic and proteolytic fermentation in the colon. The breakdown of polysaccharides inhibits proteolytic fermentation and leads to the production of saccharolytic metabolites (for example, acetate). Proteolytic fermentation results in the production of various metabolites, such as BCFAs, but also to some extent to the formation of common metabolites, such as SCFAs.

The gut microbiome is a collection of all microbes, including fungi, *archaea*, bacteria, and viruses residing in the intestinal tract.^7^ One of the key functions of the gut microbiota is the fermentation of undigested food components, such as dietary fibers and indigestible proteins, resulting in the production of various metabolites and gases that impact host metabolism. This process is generally categorized into two distinct pathways, namely saccharolytic and proteolytic fermentation (see Figure 1).^8^ Soluble dietary fibers, such as inulin, pectin, beta-glucans, and resistant starches, are the preferred energy source for the majority of the colonic gut microbiota.^6^ ^,^ ^8^ This saccharolytic fermentation yields high amounts of short-chain fatty acids (SCFAs), primarily acetate, butyrate, and propionate (approximately 95% of the total SCFA content), which have been associated with beneficial effects on gut and metabolic health.^9^ ^,^ ^10^ Alongside SCFAs, this pathway stimulates the production of other intermediary metabolites, including lactate and succinate and the gases hydrogen (H_2_), carbon dioxide (CO_2_), and methane (CH_4_).^6^

The availability of indigestible carbohydrates decreases as the fermentable substrates are moved by peristalsis from the proximal to the distal colon, leaving only a limited amount available in the distal colon.^11^ This results in an energy deficit and luminal pH becomes more neutral (as fewer SCFAs are produced). Altogether, this results in an environment where bacterial communities involved in proteolytic fermentation become more efficient, which causes the gut microbes to switch from saccharolytic to proteolytic fermentation. Proteolytic fermentation yields a wide variety of metabolites, including branched-chain fatty acids (BCFAs) and the gases ammonia (NH_3_) and hydrogen sulfide (H_2_S). Most, but not all, proteolytic metabolites are considered harmful and may have detrimental effects on gut and host health.^6^ ^,^ ^12^ For example, *in vitro* cell culture experiments with intestinal Caco-2 cells have shown that NH_3_ and p-cresol decrease gut barrier integrity and increase gut permeability at physiologically relevant concentrations.^13^ Increased p-cresol concentrations have been reported in individuals with obesity as compared with lean individuals.^14^ In humans, fecal phenol concentrations were positively associated with circulating inflammatory markers interleukin-6 and C-reactive protein.^15^ In contrast, indole, the major metabolite from tryptophan fermentation, has been shown to stimulate Glucagon-like-peptide-1 (GLP-1) secretion in murine GLUTag enteroendocrine cells via a calcium channel-dependent mechanism.^16^ Furthermore, at physiologically relevant concentrations, indoles improved epithelial cell barrier function and decreased gene expression of inflammatory markers in human HCT-8 cells.^17^ The balance between proteolytic and saccharolytic fermentation may therefore have important implications for gut and host health.^8^

Because of the general inaccessibility of the intestine in humans, our understanding of the functional activity of our gut microbiota during microbial fermentation and its interactions with host metabolism is still limited. Currently, microbial metabolites are often measured in feces, which are measured long after actual fermentation takes place, whereas techniques that could provide more detailed information at the colonic level are highly invasive.^9^

Intestinal gas production and composition, however, offers a tangible and real-time window into these processes. In humans and animals, H_2_ and CH_4_ are produced mainly during the fermentation of carbohydrates and are believed to be solely produced by bacterial metabolism, whereas the trace gas H_2_S may be reflective of proteolytic fermentation.^18^ Moreover, not much is known about the effect of these gases on the gut and host metabolism. Measurements of gut microbial-derived gas volume and microbial gas composition provide a unique opportunity to increase our understanding of the gut microbiota host–metabolism axis, as these excreted gases present a non-invasive measurement that reflects gut microbial fermentation activity.

In this review, we evaluate the concentrations of several important gut metabolites and gases. In addition, we provide an overview of various techniques to measure intestinal gas excretion and discuss their potential as screening tools or biomarkers reflecting microbial fermentation. We then discuss the pathways involved in the regulation of intestinal gas homeostasis and the potential of intestinal gases to affect host metabolism. Lastly, the potential value of intestinal gas measurements to increase our understanding of the gut‒host metabolism axis and as a basis for personalized nutrition will be discussed.

## Concentrations of microbial metabolites and gases in humans

Sudden death victims provided opportunities to measure both SCFA concentrations in the intestinal lumen and circulation simultaneously.^10^ ^,^ ^11^ In the intestinal lumen, total SCFA concentrations were mostly low in the small intestine (<1 mmol/kg luminal content) and peaking at 13 ± 6 mmol/kg in the ileum (which is in close proximity to the proximal colon). In contrast, the intraluminal concentrations of SCFAs in the proximal colon were approximately tenfold higher at 131 ± 9 mmol/kg, and the concentrations decreased gradually towards the distal parts of the colon to 100 ± 30 mmol/kg.^10^ The ratio of the main SCFAs acetate, propionate and butyrate was approximately 3:1:1 in the intestinal lumen.^6^ ^,^ ^10^ ^,^ ^11^ Colonic concentrations of succinate are relatively lower as compared with SCFAs, peaking at 3.1 ± 19 mmol/kg in the proximal colon and decreasing towards the distal colon.^10^ ^,^ ^19^ The reason that succinate concentrations are relatively low as compared to SCFAs is presumably because succinate serves as an important substrate for the production of propionate in bacterial cross-feeding.^6^

The amounts of SCFAs reaching the circulation are significantly lower when compared with their colonic concentrations. Acetate concentrations are reported at systemic concentrations between 5 μmol/l and 220 μmol/l, whereas butyrate and propionate circulatory concentrations fall in a range between 3–8 μmol/l and 7–10 μmol/l, respectively.^6^ ^,^ ^9^ ^,^ ^10^ This is because butyrate serves as a major energy source for colonocytes.^20^ ^,^ ^21^ Additionally, SCFAs produced in the caecum, ascending colon and transverse colon are transported to the liver via the portal vein. In the liver, propionate is almost completely utilized as a substrate for gluconeogenesis, whereas acetate is used to a lesser extent as a substrate for lipogenesis and cholesterol synthesis, thereby reaching higher systemic concentrations.^22^ Interestingly, SCFAs produced in the distal regions of the colon are transported to the vena cava inferior, bypassing the liver and thereby reaching the systemic circulation directly.^5^ ^,^ ^6^

Fecal SCFA concentrations range from approximately 60 to 100 mmol/kg feces.^23^ The colonic concentrations of specific proteolytic metabolites have been studied to a far lesser extent. Proteolytic metabolites have previously been measured in feces, plasma, and urine. The mean fecal concentrations expressed per mmol/kg dry matter of BCFA, phenols, p-cresol and ammonia are 18.9, 2.12, 2.4, and 160.9, respectively.^6^ The phenolic compounds, indoles and phenols are rapidly detoxified by the intestinal mucosa and are subsequently excreted via the urine. Approximately 90% of phenolic compounds are excreted in the form of p-cresol. The daily excretion rates of p-cresol are reported to be between 164 and 510 μmol/d, whereas indole concentrations are found at a much lower range of 50–115 μmol/d.^23^ Moreover, in a study performed with two sudden-death individuals, the luminal concentrations of soluble protein content and its proteolytic metabolites, phenols and p-cresol, increased steadily from the ascending colon to distal colonic regions.^11^ This supports the view that proteolytic fermentation is more prominent in the distal region compared to the more proximal region of the colon.

Intestinal gas production is considered one of the most quantifiable features of microbial fermentation. According to a recent meta-analysis, the intestinal gas excretion rates on a regular diet are about 0.85 ml/min. Nevertheless, excretion rates are highly individual and can range between 0.45 and 1.63 ml/min.^24^ Colonic gas is mainly composed of nitrogen gas (mostly derived from swallowed air), H_2_, CH_4_, and CO_2_, of which only H_2_ and CH_4_ are solely produced by bacterial metabolism, largely representing saccharolytic fermentation.^25^ Other constituents are trace gases such as H_2_S, the latter mainly reflecting proteolytic fermentation, which are only found in very low quantities.^24^ ^,^ ^25^ Intestinal gas patterns are highly individual and can be related to both the gut microbiota composition and activity, gut function, such as gastrointestinal transit time, health status, and diet. In sudden death victims, the concentrations of H_2_ were highest in the proximal colon at 6–9 nmol/ml and decreased to 0.5–3 nmol/ml in the distal colon. Mean CH_4_ concentrations steadily increased from the proximal colon at 4.1 μmol/ml to 12.8 μmol/ml in the distal colon.^11^

Gaining an understanding of the production and absorption kinetics of the microbial metabolites resulting from proteolytic and saccharolytic fermentation is essential to increase our knowledge of the gut microbiota–host metabolism axis. Experiments using *in vitro* (microbial) models of the colon such as the validated TNO computer-controlled, dynamic *in vitro* gastro-Intestinal Model of the colon (TIM-2) model or the Simulator of Human Intestinal Microbial Ecosystem (SHIME) model are commonly used to investigate microbial fermentation in response to dietary substrates and to investigate pathways of microbial cross-feeding.^26^ ^,^ ^27^ Nevertheless, a major limitation of these models is that they cannot account for the rapid absorption or usage of microbial metabolites by enterocytes *in vivo*.

Measuring microbial fermentation *in vivo* is also challenging due to the inaccessibility of the colon, bacterial cross-feeding, and the rapid absorption and diffusion of metabolites and gases by colonocytes, which then transfer these substances into circulation.^9^ In humans, microbial metabolites are most often measured *in vivo* in feces as they represent a non-invasive sample collection procedure. However, the majority of luminal SCFAs are quickly absorbed by colonocytes, and as a result, only approximately 5%–10% of total SCFA production is excreted in feces.^28^ Furthermore, it has been proposed that fecal SCFAs are more reflective of distal colonic SCFA production and not necessarily more proximal fermentation, which may also hold true for other fecal metabolites of microbial fermentation.^29^ Measurements of fecal SCFAs as biomarker for overall colonic activity should therefore be interpreted with caution.

Fecal microbial metabolites are the residue of production, cross-feeding and absorption and are measured long after actual fermentation took place.^9^ An elegant study in mice using cecal-infused isotopically labeled SCFAs showed that the absorption kinetics of SCFAs into circulation may be more relevant for metabolic health than colonic luminal SCFA concentrations. In this study, fluxes of SCFAs were correlated with body weight, fat mass, triglycerides, fasted glucose and insulin levels, and HOMA-IR, whereas cecal SCFA concentrations did not correlate with any of these markers.^30^ In addition, it has been revealed that circulating, but not fecal, SCFAs are related to insulin sensitivity, lipolysis and circulating GLP-1 levels in humans again, demonstrating that fecal SCFAs may not be a good biomarker for microbial effects on host metabolism.^31^ A major benefit of excreted intestinal gas measurements is that it is a direct marker of gut microbial fermentation that can be acquired non-invasively.

Particularly noteworthy is a recently published study that used microbial community-scale metabolic modelling models to predict SCFA fluxes.^32^ Using microbiome data of 3,129 deeply phenotyped individuals, it was revealed that predicted butyrate fluxes were negatively associated with various health parameters and plasma markers, including BMI (body mass index), fasting glucose, insulin resistance, blood pressure, C-reactive protein, and low-density lipoprotein cholesterol. On the other hand, none of these cardiometabolic and inflammation markers were associated with circulating butyrate. These findings could be explained by the rapid turnover of butyrate by enterocytes, further highlighting the limitations of the current approaches to measure microbial fermentation.

## Measuring fermentation gases as biomarkers of microbial activity?

Ever since interest in the measurement of intestinal gases arose as early as the mid-1800s, various direct and indirect techniques have been developed to measure intestinal gas volume and composition.^33^ Discussing all the different techniques and sampling methods that can be employed to measure microbial gas production is beyond the scope of this review. Direct measurements are still considered the golden standard; however, indirect measurements are used most often nowadays due to their non-invasive nature.^25^ Within this review, we will focus on the non-invasive methods that can be employed to measure gut microbial activity based on the production and composition of intestinal gases.

### Breath sampling

The most performed measurement of intestinal gases is in exhaled breath air, as it is quick and a relatively easily reproducible non-invasive measurement. During fermentation, intestinal gases are produced and accumulate in the colon. A part of these gases diffuses over the intestinal epithelium into the bloodstream from where it is excreted through the lungs.^33^ During a typical breath test, a carbohydrate load or fermentable substrate such as lactulose is provided, and over a period, subsequent measures are performed.^25^ Breath composition is generally analyzed using gas chromatography-mass spectrometry (GCMS). H_2_ is the most commonly measured intestinal gas, followed by CH_4_ , which is a marker of carbohydrate fermentation since these gases are believed to be solely derived from microbial fermentation. These breath tests are routinely used in clinical practice to test for gut diseases such as small intestinal bacterial overgrowth (SIBO), glucose or fructose malabsorption, and irritable bowel syndrome (IBS).^25^ ^,^ ^33^

Overall, breath sampling is a very accessible non-invasive technique to measure microbial activity and human metabolism. Nevertheless, a major drawback of breath measurements is the lack of standardization and consensus between studies.^25^ For example, the duration, sampling time points and amount of substrate provided may differ per study. Furthermore, breath sampling only provides snapshots, which lack the information that real-time continuous measurements could provide.

### Stable isotope breath analyses

Another type of breath testing involves the use of carbon isotopes. Breath testing has been performed using stable and unstable isotopes, of which the first is often preferred owing to its lack of radioactivity.^34^ Carbon-13 (^13^C), for example, is a stable isotope representing 1.109% of the carbon atoms in ambient air, with ^12^C representing almost all others. Substrates can be labeled with ^13^C to trace the kinetics of specific dietary substrates, such as glucose, palmitate or dietary fibers. The metabolism of ^13^C substrates leads to full oxidation to the production of ^13^CO_2,_ which is excreted in the breath on top of the naturally occurring ^13^CO_2._ ^25^ ^,^ ^35-38^ Although relatively expensive, the benefit of using stable isotopes is that this technique allows for the tracing and quantification of metabolites derived solely from the provided labeled substrate. Nevertheless, this remains difficult as there is a loss in the tricarboxylic acid cycle which needs to be individually corrected.^39^ Isotopically ^13^C-labelled fibers have been used to trace the metabolic fate of dietary fibers in both animal and human studies.^40^ ^,^ ^41^ In more detail, the effects of a single dose of wheat bran on metabolic parameters showed no differences in the capacity to ferment ^13^C-labelled inulin in healthy men and women at different particle sizes,^40^ while ingestion of a high-fat mixed meal with ^13^C-labelled inulin increased circulating ^13^C-SCFAs and breath ^13^CO_2._ ^42^ A recent study investigated the effects of ^13^C-labeled wheat bran biscuits in healthy individuals and observed a drastic difference in ^13^CH_4_ production in high-CH_4_ producers versus low-CH_4_ producers.^43^

### Breath volatile organic compound analysis

The human breath contains approximately 200 different volatile organic compounds (VOCs), which partly reflect endogenous, but also microbial metabolism. Several studies have related breath VOCs to health-related parameters.^44^ For example, breath acetone has been proposed as a potential biomarker of diabetes.^45^ More recently, this approach has also been applied to study the changes in VOC profiles to various foods and food components, including dietary fibers.^44^ ^,^ ^46-48^ A pilot study showed that the ingestion of a high-fiber diet versus a low-fiber diet for two weeks resulted in a decrease in fasting breath 2-methylbutanoic acid. Furthermore, postprandial breath 1-propanol was lower following high-fiber treatments.^46^ Even the ingestion of a single dose of chitin-glucan fiber has been shown to alter the breath VOC composition. A single dose of chitin-glucan increased breath butyrate peaking after 6 h of intake, whereas expired breath propionate was decreased throughout the complete test when compared to maltodextrin ingestion. Furthermore, expired methanol, triethylamine, ethane, pentane, 2,3-butadione and 3-hydroxybutanone were altered.^48^ Moreover, VOCs have also been correlated to specific gut bacteria species.^47^ ^,^ ^48^ Changes in breath VOCs have also been observed in fifteen healthy individuals after a three-week chitin-glucan supplementation, as breath VOCs butyrate, caproic acid, butanol, ethanol, phenol, CH_4_, pentane, triethylamine, 3-hydroxybutanone and 2,3 butanedione were significantly altered after a standardized breakfast.^47^ Interestingly, no changes in breath VOCs have been observed in studies that supplemented milk oligosaccharides and pectin.^49^ ^,^ ^50^

Despite quantitative interindividual and intraindividual variation of breath VOCs, measuring breath VOCs holds promising potential to further increase our understanding of the metabolic interactions between nutrition, the microbiota and host metabolism, as it can provide a non-invasive snapshot of the complex dynamic responses between nutrients and the gut microbiota.

### Intestinal gas capsules

The use of indigestible capsules is a relatively new technique to measure microbial fermentation. These capsules can measure the local contents of the intraluminal environment and therefore directly measure colonic gas concentrations. Furthermore, these capsules can indicate the location of fermentation in the colon via O_2_ concentration measurements.^51^ However, a limitation of this technique is that the sensors cannot be used to measure fermentation patterns over a longer period, as the sensors are excreted once they fully pass through the colon. In addition, although these sensors can measure intestinal gases directly, they can only do this in one specific location and do not provide insight into total gas production.

### Whole room calorimeters

Several earlier studies have used respiration chambers to measure excreted intestinal gases. Whole-room calorimeters, also known as respiration chambers, are airtight chambers with an airlock and a constant supply of ambient air. Respiration chambers are mostly used to measure changes in energy expenditure over longer periods through measurements of O_2_ consumption and CO_2_ production.^52^ In these studies, respiration chambers were used to study the effects of dietary and pharmacological interventions on gas production during fermentation.^53-56^ Contrary to methods that collect solely rectal or breath gas samples, this method allows for the measurement of total fermentation gas excretion (breath and flatulence). All studies except for one determined intestinal gas concentrations by collecting samples of outgoing air using gas entrapment systems e.g. Douglas bags. The collected gas samples were later analyzed using electrochemical cells and flame-ionized detectors.^54-56^ In one study, a respiration chamber was equipped with sensors to measure fermentation gases, allowing for direct room measurements.^53^ These studies reported mean excretion rates of H_2_ between 35 and 782 ml/24h and CH_4_ excretion rates between 86 and 306 ml/24h.^53^ ^,^ ^54^ The size of the whole-room calorimeters ranged between 1.4 m^3^ and 14 m^3.^ ^53-56^ The high variability in observed excretion rates can be attributed to differences in the studied populations—such as healthy individuals and those with IBS—as well as the various interventions applied, including fiber-free diets, supplementation with different types of fibers, and antibiotic treatment.

These studies also showed for the first time that the proportion of H_2_ excreted from the breath is highly dependent on the total gas excretion rates, as during high excretion rates a relatively lower percentage of fermentation gas was excreted via the breath.^54^ ^,^ ^55^ These data importantly suggest that the use of breath testing is less accurate to measure (total) microbial fermentation, as these may not accurately reflect total fermentation gas excretion, especially during periods of high microbial activity. Additionally, it was shown that more slowly fermentable fibers yield a lower total excretion rate of H_2_ compared with rapidly fermentable fibers, and similar trends were observed for CH_4_ production.^55^

The application of real-time fermentation sensors in an indirect calorimetry system for mice showed that even the difference between low- and high-digestible starches resulted in differential effects on H_2_ measurements,^57^ supporting the view that sensitive measurements of H_2_ are applicable, and future studies need to provide detailed insights into the effects upon fiber interventions. Of note, it was recently shown that 24-h H_2_ production was only seen in standard mice, not in germ-free mice, underscoring the sensitive measurements of gut microbiota activity in such a system.^58^ Recently, a whole-room calorimeter has been developed and validated that is capable of measuring CH_4_ continuously in humans.^59^ The current use of such a chamber is still limited, as CH_4_ is only one of the intestinal gases produced during saccharolytic fermentation. A system combining indirect calorimetry and CH_4_ measurements with other important intestinal gases, such as H_2_ and H_2_S, will allow us to further understand the relation between proteolytic and saccharolytic fermentation and host metabolism in humans. We are currently performing a validation study of a respiration chamber that is capable of calorimetry and measurement of H_2_, CH_4_, H_2_S.^60^ In addition, this chamber will be equipped with a ^13^CO_2_ that will allow us to track the kinetics of fermentation using isotopically labeled dietary fibers.

## Intestinal gas homeostasis and its impact on metabolic health

**Figure 2.**
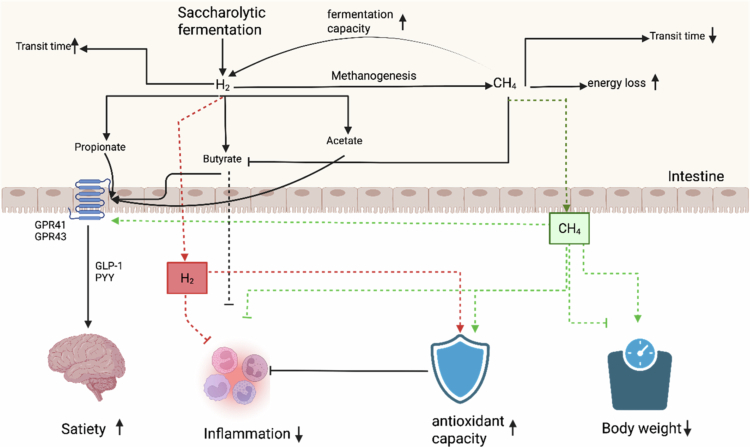
Interplay between H_2_ and CH_4_ production and their impact on host metabolic pathways. The black arrows indicate direct colonic-mediated effects from intestinal gases, the red lines indicate direct effects of H_2,_ and the green arrows indicate direct effects of CH_4_ on host physiology. H_2_ is derived from saccharolytic fermentation and converted into SCFA and CH_4_. The conversion of H_2_ to CH_4_ causes energy loss and reduces partial pressure, allowing fermentation to continue and shifting fermentation from butyrate to acetate. SCFA can affect satiety through GPR activation. In the colon, H_2_ decreases the transit time, whereas CH_4_ increases the transit time. H_2_ and CH_4_ may also directly beneficially affect the host by improving satiety, inflammation, antioxidant capacity and body weight through direct and indirect mechanisms. H2; hydrogen, CH4; methane, GPR; G protein-coupled receptors, GLP-1; glucagon-like peptide-1; PYY, protein YY.

**Figure 3.**
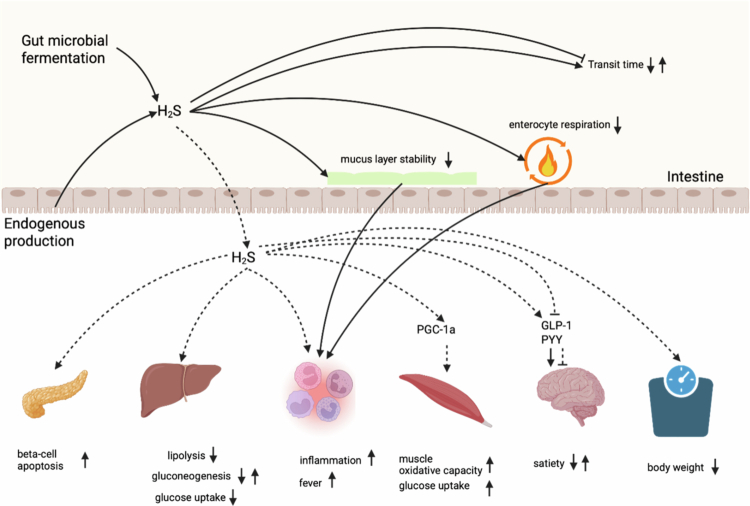
Proposed mechanisms through which H_2_S may directly and indirectly affect metabolic health. The black lines represent the effects of H_2_S mediated from the colon, where the dotted lines represent the direct peripheral effects of H_2_S on metabolism. H2S can be derived from endogenously and from fermentation. In the colon, H2S disrupts mucus layer stability, enterocyte respiration and has mixed effects on gastrointestinal transit times. H_2_S may also directly affect the host by increasing beta-cell apoptosis, altering liver metabolism, increasing inflammation, and improving muscle oxidative capacity, satiety signaling and body weight. H_2_S; hydrogen sulfide, PGC-1a; Peroxisome proliferator-activated receptor gamma coactivator 1-alpha, GPR; G protein-coupled receptors, GLP-1; Glucagon-Like-Peptide-1; PYY, Protein YY.

The physiological and clinical utility of intestinal gases lies in their dual role as both markers of fermentation and their instrumental role in the physiological process of gut microbial fermentation (see Figure 1). Moreover, rather than being inert intermediaries and end-products, H_2_, CH_4_ and H_2_S may interact directly with host tissues, influencing motility, satiety, and inflammatory pathways and thereby influencing host metabolic health (see Figures 2 and 3). Understanding the homeostasis of these gases requires an integrated view of their microbial origins and their subsequent physiological effects on the host.

### Hydrogen and carbon dioxide: the primary products of saccharolytic fermentation and host metabolic health

Bacterial breakdown of undigested carbohydrates is the major source of H_2_ and CO_2_ gas in the colon. It is important to note, however, that while excreted H_2_ is solely derived from bacterial metabolism, CO_2_ is also a main product excreted from host metabolism. In comparison to the amount of CO_2_ produced by the host, the amount of CO_2_ produced by the microbiota is substantially less and depends largely on substrate availability in the colon. Moreover, excreted CO_2_ is partly derived from swallowed air and produced from chemical reactions in the stomach and small intestine. These factors make CO_2_ less suitable as a marker of fermentation, as this can only be quantified using tracers such as 13-C labeled fibers. Nevertheless, this would reflect both ^13^CO_2_ production from the colon and the metabolism of microbiota-derived energy substrates such as SCFA by the host.^42^

H_2_ is an end-product produced by many primary fermenters and plays an important role in the regulation of gut homeostasis as an electron sink, allowing fermentation to continue.^61^ Approximately 70% of all gut microbiota possess the genetic capability to produce H_2._ ^62^ The majority of the bacteria containing H_2_-generating enzymes are part of the *Bacteroidetes* and *Firmicutes* phyla, which represent approximately 85% of total gut populations in the colon.^63^ Based on *in vitro* culture experiments, it is hypothesized that *Firmicutes* are the major contributors to H_2_ production in the colon.^33^

Crucially, CO_2_ is generated alongside H_2_ during these fermentative processes, primarily through the decarboxylation of pyruvate. The main pathway responsible for H_2_ production is the reoxidation of reduced pyridine and flavin nucleotides to maintain redox balance. Additionally, H_2_ can be produced by the cleavage of pyruvate into formate or by the generation of pyruvate by ferredoxin oxidoreductase and hydrogenases.^25^ ^,^ ^33^ ^,^ ^64^ The accumulation of H_2_ would result in a partial pressure which would halt further H_2_ production by inhibiting the regeneration of flavin and pyridine nucleotides.^33^ To prevent this and continue fermentation, H_2_ concentrations are reduced by two pathways: Firstly, by excretion as flatus and from the lungs in the breath.^55^ Secondly, through the respiration of cross-feeding hydrogenotrophic (H_2_-utilizing) bacteria.^33^ ^,^ ^65^ These bacteria can be classified into three main groups based on the end-products of their metabolism: methanogenic *archaea*, which is responsible for the production of CH_4_, sulfate-reducing bacteria using free sulfate as an electron acceptor, resulting in the production of H_2_S, and acetogenic bacteria producing acetate predominantly via the Wood-Ljungdahl pathway. It is not yet known to what extent each of the H_2_ reductive pathways contributes to the maintenance of H_2_ balance in the colon and to what extent these pathways may differ per individual. Furthermore, it is not clear how hydrogenotrophs can influence each other in composition and/or activity.

#### Preclinical studies on hydrogen and metabolic health

The homeostasis described above directly impacts host physiology, as H_2_ affects the host through the modulation of gut microbial activity.

A recent microbial *in vitro* experiment demonstrated that the incubation of several abundant butyrate-generating bacteria of the human colon at increasing H_2_ concentrations shifted microbial fermentation production from acetate to butyrate and lactate.^66^ Alternatively, although current evidence is limited, H_2_ yielded from the fermentation of dietary substrates such as dietary fibers may also affect host metabolism directly via systemic diffusion. A rodent study revealed that in male Sprague–Dawley rats, supplementation with fructo-oligosaccharides from 0%–5% of the total diet dose-dependently increased H_2_ concentrations in the portal vein, liver, adipose tissue, and abdominal cavity. Furthermore, supplementation with inulin and fructooligosaccharides for fourteen days increased adipose tissue and portal H_2_ concentrations approximately 10 to 15 fold versus the control treatment.^67^ However, the direct effects of gut-derived H_2_ on host metabolism remain difficult to isolate, as it is a primary metabolite in the production of SCFAs, CO_2_ reduction (acetogenesis), which independently affect metabolic health.^9^ ^,^ ^68^

#### Hydrogen supplementation

Over the past years, accumulating evidence has shown (non-fermentation derived) H_2_ as a potential therapeutic gas with potent anti-apoptotic, anti-inflammatory and antioxidant properties (see Figure 2). Here, we briefly discuss these findings. H_2_ was given in the form of injections, gas inhalations, and H_2_-enriched water intake in patients with a wide variety of diseases.^69^ The effects of H_2_ supplementation have also been investigated in the setting of obesity, T2D and related metabolic disorders in both *in vitro* models and in clinical settings. These studies have been described extensively in a recent review.^70^ In clinical trials, supplementation with H_2_ has predominantly been provided using H_2_-enriched water. Supplementation resulted in consistent beneficial effects on markers of glucose metabolism in individuals with T2D, impaired glucose tolerance, MAFLD and metabolic syndrome.^70^ Although these studies are promising, the sample sizes of most of these studies were relatively small, and study durations are short to medium term, mostly at 4–8 weeks.

Interestingly, supplementation of H_2_-enriched water was also shown to influence gut microbiota composition and function in rodents and piglets (see Table 1).^71-74^ For example, in a rodent model of Parkinson's disease versus controls, H_2_-enriched water increased the relative abundance of several butyrate-producing gut bacteria, increased butyrate concentrations and improved gut barrier function.^73^ There is some evidence indicating that H_2_-enriched water supplementation may also influence the human gut microbiota; however, this should be further investigated before firm conclusions can be drawn.^75^ ^,^ ^76^ Additionally, due to the high variability in H₂ production, establishing reference ranges for gut-derived H₂ remains unavailable, making it difficult to determine if H_2_ produced in microbial fermentation will result in similar effects.

**Table 1.** The effects of hydrogen on metabolic markers based on current preclinical and *in vivo* evidence.

Preclinical studies			
First author	Model/population	Intervention/exposure	Key findings
Campbell^66^	*In vitro* incubation of synthetic gut microbiota	Increasing H_2_ concentrations	Shift fermentation from acetate to butyrate and lactate production
Naomichi^67^	Male Sprague–Dawley rats	FOS (0%–5%) for 14 days	↑ H_2_ in adipose tissue and portal H_2_ concentrations
Xie^71^	Male Sprague–Dawley rats	Hydrogen-enriched water or hydrogen gas inhalation for 6 months	↑ abundance of *Lactobacillus*, *Ruminococcus*, *Clostridium XI*, ↓ in *Bacteroides*. Hydrogen inhalation ↓ *Blautia* and *Paraprevotella abundance*
Higashimura^72^	C57BL/6N mice	4 weeks of hydrogen-enriched water	↓ serum LDL and ALT ↑ cecal isovalerate, propionate and isobutyrate
Bordoni^73^	Parkinson's Wistar rat model	Hydrogen-enriched water	↑relative abundance butyrate producers ↑ gut barrier function ↓liver inflammation
Zheng^74^	Female piglets on a Fusarium-contaminated diet	Hydrogen-enriched water with lactulose	↑ H_2_ in the stomach, duodenum, colon and caecum
*In vivo studies*			
Xie^70^	Systematic review, 13 clinical trials 22 rodent studies	H_2_ supplementation	↑ improved inflammatory and metabolic markers

### Methane: microbial origins and syntrophic dynamics in relation to metabolic health

Originally, CH_4_ was considered a biologically inactive gas. However, more recent data indicate that CH_4_ may function as an important neurotransmitter and could possess important bioactive properties. Nevertheless, data regarding the relationships between CH_4_- and CH_4_-producing microbes and metabolic health are limited and are currently inconclusive. Understanding its potential impact on host metabolic health requires a combined look at its microbial synthesis and its systemic effects.

CH_4_ is an end product of saccharolytic fermentation yielded by methanogenic *Archaea* in the colon. These *archaea* use CO_2_ in combination with H_2_ and/or formate as electron donors to produce CH_4_. The main CH_4_-producing *archaeon* that is observed in humans is *Methanobrevibacter smithii*. Other CH_4_-producing bacteria that have been found in human feces include *Methanospaera stadtmanae* (which reduces formate with H_2_) and *Methanomassilicoccus luminyesis,* however, the presence and prevalence of these species are far less as compared with the *Methanobrevibacter smithii.* ^77-79^ Methanogens consume H_2_, thus, they depend on primary fermenters that break down polysaccharides for sufficient H_2_ production, as they are unable to generate H_2_. The populations of CH_4_-producing *archaea* are higher in the distal compared to the proximal part of the colon (0.3% in the proximal colon compared with 5% in the distal colon, in sudden death victims).^11^ This is most likely because methanogens are considered to be most efficient at a neutral pH.^80^

While the presence of methanogens is near-universal in humans based on fecal samples, all humans likely produce CH_4_ to a certain extent.^77^. Nevertheless, the reported presence of CH_4_ in human breath is highly variable, as it varies from 34%–60% in Western populations. In non-Western cultures that still consume more traditional diets and thus typically ingest higher amounts of dietary fibers, the presence of CH_4_ in breath is higher and varies between approximately 72%–87%.^81^ Although it is unclear what causes the high variability in breath CH_4_ presence, age seems to be an important factor, given that CH_4_ is not present in the breath of infants and generally seems to become present during adolescence.^81^ It is also debatable whether measuring breath CH_4_ is an accurate marker of CH_4_ production, as it was reported in a human study that in some individuals, CH_4_ could be detected in rectal gas samples but not in breath.^82^ Interestingly, using a high-precision method to detect breath CH_4_, it was shown that all volunteers (*n* = 122) exhaled CH_4_. However, expired CH_4_ was often lower than the usually detectable values, indicating that, in fact, all humans produce and excrete CH_4._ ^83^ This further highlights the necessity of highly accurate measurement techniques to increase our understanding of the contribution of CH_4_ to host health.

#### Preclinical studies on methane and metabolic health

The impact of CH_4_ and its producers on energy homeostasis is a subject of significant debate. Early experiments using gnotobiotic mice elegantly showed that the cocolonization of *B. thetaiotaomicron*, a common H_2_-producing species, with *Methanobrevibacter smithii* drastically enhances the fermentation of fermentable fibers in the colon. In contrast, germfree, monoassociated or biassociated models with *Desulfovibrio*, an H_2_S-producing species, did not result in enhanced fermentation capacity. The synergistically increased fermentative capabilities of the combined *B. thetaiotaomicron* with *Methanobrevibacter smithii* resulted in increased acetate production, increased liver triglycerides and energy storage, as reflected by the epididymal fat pad mass.^84^ This suggests that, by consuming H_2_ and CO_2_, methanogens drive the fermentation process toward a higher energy yield for the host (Figure 2).

In line, a recent *in vitro* study using synthetic human gut microbial communities also observed that the addition of methanogens to their model stimulated acetate production at the expense of butyrate.^66^ Moreover, mice with obesity were shown to have higher cecal contents of methanogens compared with their lean littermates.^85^ Furthermore, CH_4_ may influence metabolism by stimulating the release of GLP-1. Another mouse study revealed that methanogens were increased in fecal extracts from mice following a high-fat diet for 2 weeks when compared with chow-fed mice. Additionally, a positive correlation between circulating GLP-1 levels and fecal methanogen content during an oral glucose test (OGTT) was observed in these mice. Treatment of murine and human epithelial cells with CH_4_ enhanced the release of GLP-1 and increased the level of intracellular cAMP.^86^ Interestingly, GLP-1 stimulation was greatest in human cells at CH_4_ concentrations within the expected physiological range.^86^ The overall impact on metabolic health remains ambiguous (Figure 2 and Table 2). While an increase in methanogens may enhance energy harvesting, potentially contributing to fat storage in rodent models, it is important to note that CH_4_ itself is not metabolized as an energy substrate in humans or other mammals. This might mitigate its impact on total energy yield, similar to observations in ruminants, where CH_4_ production represents an energy loss of 2%–12%^87^ ^,^ ^88^

CH_4_ has also been proposed to influence host metabolism through its potential to serve as a molecule with potent anti-inflammatory, anti-apoptotic and antioxidant effects. For example, in a rodent model with IBS, it was shown that 10 ml/kg CH_4_-enriched saline *vs* vehicle given by *i.p.* injections drastically reduced the colonic mRNA and protein expression of inflammatory cytokines, together with reduced colonic apoptosis and increased anti-apoptotic markers in colon tissue.^89^ Of note, these data should be interpreted with caution, as these findings were only present with a CH_4_ concentration largely exceeding the assumed physiological CH_4_ concentrations in the colon.

#### Human studies on methane and metabolic health

Human data indicating a role of CH_4_ production on host metabolism is mainly derived from observational studies, and these results are inconclusive (Table 2).^90-100^ In detail, an early study observed that breath CH_4_-positive individuals had a lower prevalence of obesity measured by skinfold thickness when compared to individuals who did not excrete CH_4_ in their breath. Additionally, an inverse relationship between breath CH_4_ and skinfold thickness was observed.^91^ A large cohort study comprised of 1647 individuals with functional gastrointestinal disorders reported that lean individuals had higher breath CH_4_ levels after the intake of fructose and lactose when compared to individuals with overweight and obesity. Similar effects were observed regarding waist circumference following the intake of fructose but not lactose. Furthermore, peak breath CH_4_ concentrations greater than 3 ppm (0.0003%) were associated with a lower BMI and waist circumference, independent of breath H_2_ production.^98^ A study including 94 individuals reported decreased breath CH_4_ in individuals with obesity when compared to lean individuals.^99^ In a Japanese cohort study consisting of 1,033 individuals, fasting breath CH_4_ was also inversely correlated with visceral adiposity and waist circumference.^92^ Interestingly, the relative abundance of *Methanobrevibacter smithii* also seems to be enhanced in underweight individuals when compared to individuals with normal body weight or overweight.^101^ ^,^ ^102^ The strongest evidence regarding a potential relationship between methanogens and body weight comes from a recent meta-analysis comprised of fecal samples from 1,821 individuals from 10 studies. This analysis concluded that the relative abundance of methanogenic species was inversely related to BMI.^94^

A study with a small sample size (*n* = 9) showed that individuals with morbid obesity had a higher relative fecal abundance of methanogenic *archaea* when compared with both individuals with morbid obesity who underwent gastric bypass surgery eight to fifteen months before fecal sampling and lean individuals. The latter study sparked interest in the role of methanogenic *archaea* in humans.^95^ In line with this finding, another cross-sectional study observed that the relative abundance of fecal *archaea* and SCFAs were increased in individuals with overweight or obesity when compared with overweight and obese individuals after surgery-induced weight loss and when compared with normal weight individuals.^97^ Nevertheless, the results of this study should be interpreted with caution, as the sample size of this study was relatively small (*n* = 20) and there was considerable heterogeneity in age and BMI amongst and within the studied population. In the KOALA birth cohort, it was observed that in 295 children aged 6–10 years, a higher stool methanogenic *archaea* content, particularly *M. smithii*, was related to increased BMI z-scores with age.^96^ Others have also observed a higher BMI in individuals with a breath CH_4_ positive status (>3 ppm within 90 min or 120 min after ingestion of lactulose) and a higher BMI and fat percentage in individuals who were both CH_4_ and H_2_ producers (CH_4_ positive and H_2_ ≥ 20 ppm) following ingestion of lactulose.^90^ ^,^ ^100^

The reason for this seemingly ambiguous relationship regarding obesity and CH_4_ production in these observational studies may be related to differences in study design, sampling methods and study populations. Particularly in studies that investigated the dynamic response of intestinal gas production following the ingestion of a fermentable substrate, the duration of the gas measurements and the type of fermentable substrate may be important. For example, the two aforementioned studies that observed a positive correlation between markers of obesity and breath CH_4_ had a measurement duration of 90 to 120 min as compared with a duration of five hours in the study performed in 1647 patients with functional gastrointestinal disorders that observed an opposite relationship.^90^ ^,^ ^98^ ^,^ ^100^ These shorter measurement durations may be more reflective of the small intestinal microbiome or those with a rapid gastrointestinal transit time, highlighting the importance of measuring microbial fermentation over a prolonged period to get a clear view of fermentation gas patterns and their relation to human body weight and composition.^98^ Nevertheless, when considering the overall strength and quality of these studies, particularly the large recently published meta-analysis, it seems most likely that higher breath CH_4_ is inversely associated with body weight.^94^

A small cross-sectional study comprised of five breath CH_4_-producing and 15 non-CH4-producing individuals observed that breath CH_4_-positive individuals displayed an increased glucose area under the curve during an OGTT when compared with breath non-CH_4_ producers.^103^ To our knowledge, there is only one intervention study to date that purposefully tried to modulate colonic CH_4_ production. In this study, 11 individuals with prediabetes received a 10-day antibiotic treatment with neomycin and rifaximin to reduce breath CH_4_ concentrations. In individuals in whom breath CH_4_ was reduced following antibiotic treatment, insulin and glucose levels during an OGTT were lower when compared with individuals in whom breath CH_4_ production remained similar following treatment. Interestingly, the fecal methanogen content was significantly lower after antibiotic treatment in all individuals, independent of CH_4_-exhalation levels.^104^ Furthermore, antibiotics disrupted more than only methanogens, and the observed effects could therefore have been caused by the disruption of other bacterial and *archaeal* communities or indirectly via intestinal barrier function alterations.^105^

Overall, these data indicate that CH_4_ production may play an important role in the regulation of host metabolism by increasing the efficiency of microbial fermentation by acting as a sink for H_2_ produced by many primary fermenters, resulting in increased SCFA production, modulating gastrointestinal transit time, regulating inflammatory status, and by stimulating GLP-1 secretion of enteroendocrine cells via a cAMP-regulated mechanism. Data from human studies remain controversial, and well-controlled human studies investigating the direct relationship between CH_4_ gas production and/or exhalation and metabolic health are eagerly awaited. Additionally, studies investigating how specific CH_4_ production patterns over prolonged periods of time are related to metabolic health are warranted, as this may explain some of the controversies observed in these human studies.

**Table 2.** The effects of methane on metabolic markers based on current preclinical and *in vivo* evidence.

Preclinical studies				
First author	design	Model/population	Intervention/exposure	Key findings
Samuel^84^		Gnobotic NMRI/KI mouse *Cocolonization with Methanobrevibacter smithii, Bacteroides thetaiotaomicron and D. piger*	*Fructans*	Cocolonization with M.smithii and b.*thetaiotaomicron↑ energy yield, acetate and epidymal fat mass*
Campbell^66^		*In vitro* incubation synthetic gut microbiota	Addition of *m. Smithii* to a synthetic gut microbiota	↑acetate production ↓ butyrate production
Turnbaugh^85^		Ob/ob, ob/+ and +/+ mice littermates	Not applicable	Ob/ob mice ↑ relative abundance of methanogenic *archaea* compared to *ob*/+ and +/+ littermates
Laverdure^86^		C57Bl6 male mice, Mouse (GLUTag) and human (NCI‐H716) L cells	14 weeks high-fat diet, 48-h incubation with CH_4_	High fat-diet ↑ fecal methanogen content and ↑ GLP-1 secretion, Incubation with CH_4_ ↑GLP-1 secretion
Shen^89^		C57BL/6J mice IBD model	CH_4_-enriched water	↓ oxidative stress ↓apoptotic cells pro/anti-inflammatory markers ↓TLR4, MyD88, p-NF-κB p65, p-IKKαβ, and p-IκBα and ↑ IL-10, p-JAK1, and p-STAT3
*In vivo studies*				
Haines^91^	Cross-sectional	1267 men 604 women		Breath CH_4_ was inversely associated with skinfold thickness in males and in females
Wilder-Smith^98^	Cross-sectional study	1647 patients with functional gastrointestinal disorders.		Higher breath methane was associated with ↓ BMI and ↓ waist circumference
Fernandes^99^	Cross-sectional	52 lean and 42 overweight or obese individuals		↑ Breath CH4 was greater in lean individuals
Ozato^92^	Cross sectional	1033 individuals		Inverse correlation between visceral fat and breath CH_4_ concentrations
Armougom	Cross-sectional	20 lean individuals, 20 obese individuals and 9 anorexic individuals		↑ Relative abundance of methanogens in anorexic individuals vs lean individuals, not versus obese individuals
Mack	Cross-sectional	Anorexic individuals (before and after weight regain) and normal-weight individuals		↑relative abundance fecal methanogens in anorexic individuals before weight gain compared to normal-weight individuals
Ruaud^94^	Meta-analyses	Fecal samples of 1,821 individuals		The relative abundance of *M. Smithii* correlated negatively with BMI
Zhang^95^	Cross-sectional	9 individuals, normal weight (*n* = 3), morbidly obese (*n* = 3), and post-gastric-bypass surgery (*n* = 3)		Relative abundance of Archaea ↑ in obese individuals compared to normal weight individuals and individuals after post-gastric bypass surgery
Patil^97^	Cross-sectional	15 individuals, normal weight (*n* = 3), obese (*n* = 3), and surgically treated obese (*n* = 3)		Individuals with obesity ↑fecal SCFAs and ↑ relative abundance of methanogenic archaea Individuals after surgical treatment of obesity ↓ fecal SCFAs, and ↓ relative abundance of methanogenic *archaea*
Mbakwa^96^	Longitudinal prospective study	472 children		Methanobrevibacter smithii colonization↑ risk of overweight and ↑ weight z-scores in children
Basseri^90^	Cross-sectional study	58 individuals with obesity (breath CH_4_ present in *n* = 12)		CH_4_-positive individuals ↑ BMI and ↑constipation
Mathur 2013^100^	Cross-sectional	792 individuals stratified as Normal, H_2_-positive, CH_4_-postive and H_2_ an CH_4_ positive		H2 and CH4-positive individuals had ↑ BMI compared to other groups and had a ↑ bodyfat%
Mathur 2014^103^	Cross sectional	20 individuals (5 methane-producers and 15 non-methane producers)		CH_4_ producers ↑ serum glucose AUC during OGTT
Mathur 2016^104^	Prospective intervention study	11 individuals with prediabetes and CH_4_-postive breath status	10-d neomycin and rifaximin treatment	CH4 eradication ↓ LDL-cholesterol, total cholesterol and ↓ insulin at *t* = 120 and glucose at *t* = 90 during an OGTT

### Hydrogen sulfide: a dual-role gasotransmitter in metabolic health

H_2_S has emerged as an important trace gas transmitter with potentially both beneficial and harmful effects on the human gut and metabolic health (Figure 3). H_2_S can be produced from three major sources: as mentioned previously, it can be generated by sulfate-reducing bacteria (SRB) using sulfur as an electron donor during fermentation.^25^ ^,^ ^65^ Secondly, fermentation of dietary proteins, particularly the sulfated amino acids methionine and cysteine, also yields high levels of H_2_S. Thirdly, H_2_S is also endogenously produced by mammalian tissues by the three enzymes cystathionine β-synthase (CBS), cystathionine γ-lyase (CSE), cystathionine γ-lyase and 3-mercaptopyruvate sulfurtransferase (MST).^106^ Gut luminal concentrations of H_2_S vary but are usually reported at 0.2–1.5 mM for rodents and up to 3.4 mM for humans.^107^ The gut microbiota may be a major regulator of the host's H_2_S availability, as it was shown that germ-free rodents have approximately 50%–80% lower tissue and circulating H_2_S when compared with controls.^108^ Interestingly, in this model, endogenous H_2_S production via CSE was also reduced, indicating that microbiota-derived H_2_S might influence the host’s H_2_S-generating enzyme activity. H_2_S also functions as a neurosignaling molecule. An elegant study showed that an increase in endogenous H_2_S synthesis induced by LPS injection in rats caused fever. This effect could be fully blocked by co-injection with propargylglycine, which inhibits the enzymes responsible for endogenous H_2_S synthesis.^109^

### H_2_S: a dual-edged sword

The role of colon-derived H_2_S in host metabolic health is currently unclear, but it appears to be highly concentration-dependent. In the intestines, H_2_S is derived from H_2_S-producing gut microbiota and, to a smaller extent, from endogenous CSE activity in colonocytes.^110^ In the colon, H_2_S serves as an energy substrate via the sulfate oxidation unit H_2_S.^110^ ^,^ ^111^ At high concentrations, however, H_2_S has been shown to inhibit colonocyte mitochondrial respiration and thus cellular energy production in rodents. This also led to increased expression of inflammatory-related genes encoding for Il-6 and i-NOS in colonocytes.^110^ ^,^ ^112^ In accordance, H_2_S reduced butyrate oxidation in isolated human colonocytes.^110^ ^,^ ^113^ Increased production of H_2_S by the microbiota has also been shown to lead to destabilization of the mucus layer in mice receiving a heme-enriched diet.^114^ Disruption of the mucus layer may lead to endotoxemia, which is associated with increased inflammation and insulin resistance.^115^

#### Preclinical studies on hydrogen sulfide and metabolic health

Preclinical studies suggest that increased H_2_S concentrations may lead to dysfunction and apoptosis of pancreatic islet beta-cells, leading to T2D (Table 3).^116^ ^,^ ^117^ Interestingly, circulating H_2_S seems to be reduced in individuals with T2D.^118^ The liver is hypothesized to be a major destination of gut-derived H_2_S through the portal vein. It is therefore assumed that the liver is subjected to high amounts of H_2_S. In the liver, H_2_S has been shown to impair glucose uptake, alter gluconeogenesis and decrease lipolysis based on cell culture and rodents.^107^ Cell culture and rodent experiments have revealed that H_2_S can also affect adipose tissue function, as H_2_S has been shown to improve adipocyte insulin sensitivity, increase adipogenesis and lipolysis-related protein expression, and reduce the protein expression of monocyte chemoattractant protein.^119^ In the skeletal muscle of mice with diet-induced obesity, H_2_S production capacity was found to be reduced, and skeletal muscle oxidation was impaired. Interestingly, the addition of cysteine or H_2_S donors (compounds such as NaHS, or Na_2_S, which degrade into H_2_S in a controlled fashion) to C2C12 myotubes improved glucose homeostasis via PGC-1α (peroxisome proliferator-activated receptor gamma coactivator 1-alpha)-dependent pathways.^120^ ^,^ ^121^

Regarding direct colonic stimulation, one study observed that sulfate donors inhibited bile acid-stimulated GLP-1 and PYY secretion in STC-1 enteroendocrine cells *in vitro.* ^122^ Providing genetically obese db/db mice with an exogenous H_2_S donor (NaHS, 80 μmol/kg) *i.p.* injected for 16 weeks reduced body weight gain and circulating triglycerides compared with vehicle-treated control db/db mice.^123^ Furthermore, decreased circulating insulin and a small reduction in blood glucose were observed in the NaHS-treated group. Nevertheless, no changes were observed in glucose tolerance. Providing the mice with NaHS also altered the gut microbiota composition, as at the phylum level, the relative abundance of Firmicutes increased, and the relative abundance of Bacteroidetes decreased.^123^ These effects can, however, not be attributed solely to gut microbial activity and H_2_S production, as endogenous H_2_S production is likely also enhanced. Also, H_2_S donors have been shown to stimulate GLP-1 secretions in murine enteroendocrine cells via a p38 MAPK-mediated mechanism.^124^ Of interest, focused on gut microbial H_2_S production, male wild-type C57BL/6 mice supplemented for 4 weeks with chondroitin sulfate, a H_2_S promoting prebiotic, in combination with a low-fiber diet, increased, among others, colonic content’s H_2_S and fecal H_2_S levels. And was accompanied by improved glucose tolerance and increased insulin and GLP-1 secretion.^124^

#### Human studies on hydrogen sulfide and metabolic health

To our knowledge, human data regarding the links between H_2_S and metabolic health is very limited, particularly with regard to gut microbial-derived H_2_S. Beyond the observation that circulating H_2_S is reduced in T2D,^118^ a small study in 36 healthy volunteers found that circulating H_2_S was positively associated with adiponectin, an adipokine that may play a crucial role in the regulation of insulin sensitivity and energy metabolism.^125^ ^,^ ^126^ In conclusion, H_2_S is an important neurotransmitter produced endogenously by enzymatic conversion in human and animal tissues, as well as by gut microbiota fermentation in the colon. H_2_S plays an important role in the regulation of gut energy homeostasis, inflammation and intestinal barrier function. Currently, most research regarding the role of H_2_S in the regulation of metabolic health has been performed in *in vitro* models focusing on the role of H_2_S production using animal and human cells. It is currently unclear to what extent H_2_S derived from microbial fermentation affects host metabolism *in vivo*, although its contributions to the host bioavailability of H_2_S may be substantial.^118^

**Table 3.** The effects of hydrogen sulfide on metabolic markers based on current preclinical and *in vivo* evidence.

Preclinical studies				
First author	design	Model/population	Intervention/exposure	Key findings
Yang^116^		Pancreatic beta-cells (INS-1E)	Stimulate endogenous H_2_S production (CSE activation)	CSE-induced H_2_S ↑ beta-cell apoptosis
Wu^117^		Zucker diabetic rats, Zucker fatty rats, and Zucker lean rats	DL-propargylglycine blocking CSE activity	CSE expression and H_2_S concentrations ↑ in diabetic Zucker rats versus Zucker fatty and Zucker lean rats PPG blocking H_2_S ↑ insulin ↓ HbA1c and glucose concentrations
Jain 2010^a^,^118^		Streptozotocin-treated diabetic rats compared with control Sprague–Dawley, high-glucose-treated human U937 monocytes	H_2_S and L-cysteine supplementation in culture medium	Fasting plasma H_2_S ↓ in diabetic rats versus normal rats H2S and L-cysteine, prevents IL-8 and MCP-1 secretion in high-glucose–treated human U937 monocytes
Parsanathan^120^		High-fat diet, obese diabetic C57BL/6J mice, Mouse C_2_C_12_ cells	L-cysteine and H2S donor supplementation	High-fat diet ↓CSE mediated H_2_S production and impaired glucose homeostasis H_2_S increased GLUT-4 and glucose uptake via PGC-1α-dependent pathways
Bala^122^		STC-1 cells	H_2_S donors	H_2_S inhibits bile acid-stimulated GLP-1 and PYY secretion
Liu^123^		*db/db* mice	16-week NaHS injections versus control	NaHS injections ↑ circulating H_2_S concentrations ↓body weight gain ↓ circulating insulin and glucose concentrations ↑ relative abundance of *Firmicutes* and ↓ relative abundance of *Bacteroidetes*
Pichette^124^		L-cells (GLUTag), C57BL/6 mice	H_2_S donors (NaHS and GYY4137), prebiotic chondroitin sulfate for 4 weeks	H_2_S donors stimulated GLP-2 in L-cells Chondroitin sulfate ↑ H_2_S concentrations in feces ↑ relative abundance of *Desulfovibrio piger* ↑ GLP-1, insulin secretion, glucose tolerance and reduced food consumption
*In vivo studies*				
Jain 2010^a^ ^,^ ^118^	Cross-sectional study	Healthy individuals (*n* = 14) and individuals with T2D (*n* = 63)		Individuals with T2D have ↑ plasma H_2_S concentrations
Jain 2012^125^	Cross-sectional	healthy individuals (*n* = 36)		Fasting H_2_S was positively correlated with adiponectin

^a^ Year of publication.

### Intestinal gases and gastrointestinal transit time and the integrity of the colonic environment

Intestinal gases yielded from microbial fermentation have been shown to affect gastrointestinal transit time, which may be an important factor in the regulation of host metabolic health by influencing nutrient availability and gut microbiota composition.^127^ An *in vitro* experiment using the guinea pig ileum revealed that H_2_ production decreases intestinal transit time, whereas CH_4_ decreased peristalsis.^128^ In humans, the presence of CH_4_ in breath has been associated with constipation according to a meta-analysis, which includes the data of 1277 individuals.^129^ It should be noted, however, that in this analysis, seven out of nine included studied populations consisted of IBS patients. In healthy individuals, those considered CH_4_ producers also tend to have increased transit times when compared to individuals who expired no detectable CH_4._ ^130-133^ Generally, H_2_S has been shown to decrease gut motility via various mechanisms, such as the opening of potassium channels, the inhibition of calcium channels and by decreasing neuronal cholinergic and tachykinergic neural activity-mediated muscular contraction.^134^ Interestingly, one study demonstrated that H_2_S exposure led to early transient excitation and late long-lasting inhibition of gut motility.^134^ ^,^ ^135^ This regulatory role of intestinal gases in transit time underscores their importance beyond being mere byproducts, as they actively shape the kinetic environment of the human gut and its subsequent metabolic outputs. Furthermore, altered gas dynamics may have structural implications for the colon; For instance, higher breath CH_4_ has previously been observed in individuals with diverticulosis.^136^ It is worth speculating whether the specific composition or total volume of these gases could also influence the incidence of diverticulitis by altering intraluminal pressure or mucosal defense mechanisms. Furthermore, the relationship between intestinal gas production and tissue permeability may extend to extra-intestinal sites. Whereas H_2_ stimulates the production of SCFAs, which may contribute to improved gut barrier integrity, specific intestinal gases, such as H2S, while essential for gut homeostasis, have been shown to improve but also disrupt intestinal barrier function by decreasing mucus layer stability at higher concentrations.^137^ ^,^ ^138^ This may facilitate the translocation of enteric pathogens, which may subsequently contribute to systemic inflammation and other pathologies in connected organs, such as metabolic dysfunction-associated steatohepatitis or intrahepatic cholangiocarcinoma (HCCC).^139^ ^,^ ^140^ Interestingly, this systemic translocation might also influence intratumoral microbial signatures; these specific microbial profiles may be relevant for predicting responses to treatment.

## Changes in intestinal gas production and metabolic health after dietary interventions

The interaction between the gut microbiota and host metabolism is multifactorial and complex. The trade-off between proteolytic and saccharolytic fermentation may be an important regulator of host metabolic health. Shifting fermentation towards more saccharolytic fermentation may be a feasible strategy to improve metabolic health and prevent the development of metabolic diseases.^6^ Diet is a major factor influencing gut composition and functionality. Dietary changes have been shown to rapidly alter gut microbial fermentation both in *in vivo* and *in vitro* studies. High-precision measurement of intestinal gas production may be used as a measurement to study the balance between proteolytic and saccharolytic fermentation directly in humans in a non-invasive fashion, thereby providing important information on the interaction of the gut microbiota with fermentable substrates. H_2_S may serve as a microbial gas marker of proteolytic fermentation, whereas H_2_ and CH_4_ are considered well-known markers of carbohydrate fermentation,^25^ although there is some overlap. It has been shown that meat intake dose-dependently increases colonic H_2_S production as measured by fecal concentrations.^141^ Furthermore, a crossover study revealed that a one-week consumption of high-fiber plant-based diets seem to stimulate H_2_S production less when compared to low-fiber meat-based diets with the same protein content.^142^ Overall, these data indicate, similar to an *in vitro* study, that dietary protein intake promotes H_2_S production, whereas dietary fiber limits H_2_S production. Interestingly, two out of the eleven healthy individuals participating in this study produced more H_2_S on the plant-based diet.^142^ This finding suggests that interactions between the gut microbiota and fermentable substrates are highly individual. Indeed, several post-hoc analysis divided participants amongst responders and non-responders to investigate which characteristics may explain variations with regard to responsiveness to interventions.^143^ Individuals with a gut microbiota harboring more protein fermenting bacteria may thus be less susceptive to treatment with dietary fibers to improve host health.^144^ Alternatively, it could be argued that these individuals might exhibit greater responsiveness after a prolonged adaptation period, as their microbiota offers a broader window for improvement.

Based on cell culture experiments, H_2_ and CH_4_ may have a strong effect on microbial fermentation and may therefore explain some of the variation in response to dietary fibers. An environment rich in H_2_ has been shown to stimulate the growth of butyrate production, whereas the addition of methanogens shifts microbial fermentation from butyrate towards acetate production.^66^ ^,^ ^94^ These results are not completely consistent as one study reported that co-culturing *Methanobrevibacter smithii* with a hydrogenotrophic acetogen actually increased butyrate production. In this experiment, however, there was no acetate in the medium, which does not reflect the environment of the colon and may thus have influenced the results.^145^

Several studies have also observed phenotypic differences with regard to intestinal gas release following the intake of fermentable substrates. In a study investigating the effects of a two-week supplementation of resistant starch in young adults, those with detectable concentrations of breath CH_4_ had lower fecal butyrate concentrations when compared to than those where CH_4_ was not detectable after the intervention. Interestingly, these differences were not observed prior to resistant starch supplementation.^66^ Different patterns in the fermentation of wheat bran have also been observed between three high-CH_4_ producing individuals and three low-CH_4_ producing individuals; as expected, CH_4_ production was higher in CH_4_-producing individuals. Additionally, plasma and stool acetate production tended to be higher in high-CH_4_ producing individuals, whereas butyrate concentrations seemed to be higher in low-CH_4_ producers.^43^ A subgroup analysis of a randomized controlled trial investigating the effects of a three-week chitin-glucan supplementation reported no change in fecal SCFA production but did reveal significant improvements in postprandial glucose after three-week fiber supplementation. This effect was only observed in individuals who had increased H_2_ production in response to a lactulose breath test at baseline. Additionally, these individuals had lower fecal methanogen abundance when compared to the low H_2_ producers. This indicates that the capacity to ferment dietary fiber, as reflected by the H_2_ response of individuals during the lactulose test, may be a predictive factor for the effectiveness of prebiotic treatments.^146^ Overall, these results indicate that there may be specific phenotypes of microbial fermentation when considering intestinal gas excretion. Understanding the patterns of microbial fermentation by means of intestinal gas excretion measurements and its relation to host metabolic health may be used to determine specific phenotypes and phenotype-specific responses to dietary interventions.

## Future perspectives and conclusion

The contributions of proteolytic and saccharolytic fermentation in the colon to metabolic health remain to be fully elucidated. Understanding the dynamics of gut fermentation requires the integration of high-precision measurements of intestinal gas excretion with comprehensive assessments of fermentation kinetics, energy metabolism, and substrate metabolism. These combined approaches provide critical insights into the gut‒host‒metabolism axis, helping to clarify how fermentation processes drive systemic metabolic outcomes. It is particularly interesting to investigate whether there are differences in the capabilities of healthy individuals to ferment dietary substrates, such as carbohydrates and proteins, as compared with those with a compromised metabolic status, such as individuals with (obesity-associated) T2D. By incorporating techniques to phenotype the metabolic profile in detail, such as via various omics analyses, may provide mechanistic insight into the gut–host metabolism axis.

Integrating advanced deep-phenotyping techniques, including various omics analyses, will be integral in providing insight into the mechanistic underpinnings of the gut microbiota–host metabolism axis.

Currently, our understanding of the trade-off between proteolytic and saccharolytic fermentation is limited owing to the inaccessibility of the colon. Over the years, techniques to measure intestinal gas production have improved, and new techniques have become available. The incorporation of breath VOC, real-time intestinal gas measurements and the use of isotopic tracers provide tremendous opportunities to monitor gut microbial activity *in vivo*. Furthermore, intestinal gases may also directly affect host metabolic health as signalling molecules, neurotransmitters, or due to their antioxidant potential; however, further research investigating this relationship is warranted, particularly in humans. To conclude, understanding the patterns of microbial fermentation and its relation to host metabolic health may ultimately be used to determine specific phenotypes and may therefore provide a basis for the development of effective (personalized) nutritional, pharmaceutical, and lifestyle-based interventions to improve gut and host metabolic health.

## Data Availability

No new data were generated or analyzed in support of this review. Data sharing is not applicable.

## Abbreviations

T2D: type 2 diabetes mellitus
SCFA: short-chain fatty acid
H_2_: hydrogen
CO_2_: carbon dioxide
CH_4_: methane
H_2_: hydrogen
BCFA: branched-chain fatty acids
NH_3_: ammonia
H_2_S: hydrogen sulfide
TIM-2: TNO computer-controlled, dynamic *in vitro* gastro-intestinal model of the colon
MAFLD: metabolic dysfunction-associated fatty liver disease
PGC-1α: peroxisome proliferator-activated receptor gamma coactivator 1-alpha
BMI: body mass index
GCMS: gas chromatography-mass spectrometry
SIBO: small intestinal bacterial overgrowth
IBS: irritable bowel syndrome
^13^C: carbon-13
VOC: volatile organic compound
SRB: sulphate-reducing bacteria
CBS: cystathionine β-synthase
CSE: cystathionine γ-lyase
MST: 3-mercaptopyruvate sulfurtransferase
HCCC: intrahepatic cholangiocarcinoma
